# Service readiness for inpatient care of small and sick newborns: what do we need and what can we measure now?

**DOI:** 10.7189/jogh.08.010702

**Published:** 2018-06

**Authors:** Sarah G Moxon, Tanya Guenther, Sabine Gabrysch, Christabel Enweronu-Laryea, Pavani K Ram, Susan Niermeyer, Kate Kerber, Cally J Tann, Neal Russell, Lily Kak, Patricia Bailey, Sasha Wilson, Wenjuan Wang, Rebecca Winter, Liliana Carvajal-Aguirre, Hannah Blencowe, Oona Campbell, Joy Lawn

**Affiliations:** 1 Maternal, Adolescent, Reproductive and Child Health (MARCH) Centre, London School of Hygiene and Tropical Medicine (LSHTM), London, UK; 2 Saving Newborn Lives, Save the Children, Washington, D.C., USA; 3 Institute of Public Health, Heidelberg University, Heidelberg, Germany; 4 College of Health Sciences, University of Ghana, Accra, Ghana; 5 USAID, Washington, D.C., USA; 6 University at Buffalo, Buffalo, New York, USA; 7 University of Colorado, Aurora, Colorado, USA; 8 University College London Hospital, London, UK; 9 Averting Maternal Death and Disability (AMDD), New York, New York, USA; 10 The DHS Program-ICF, Rockville, Maryland, USA; 11 UNICEF, New York, New York, USA

## Abstract

**Background:**

Each year an estimated 2.6 million newborns die, mainly from complications of prematurity, neonatal infections, and intrapartum events. Reducing these deaths requires high coverage of good quality care at birth, and inpatient care for small and sick newborns. In low- and middle-income countries, standardised measurement of the readiness of facilities to provide emergency obstetric care has improved tracking of readiness to provide care at birth in recent years. However, the focus has been mainly on obstetric care; service readiness for providing inpatient care of small and sick newborns is still not consistently measured or tracked.

**Methods:**

We reviewed existing international guidelines and resources to create a matrix of the structural characteristics (infrastructure, equipment, drugs, providers and guidelines) for service readiness to deliver a package of inpatient care interventions for small and sick newborns. To identify gaps in existing measurement systems, we reviewed three multi-country health facility survey tools (the Service Availability and Readiness Assessment, the Service Provision Assessment and the Emergency Obstetric and Newborn Care Assessment) against our service readiness matrix.

**Findings:**

For service readiness to provide inpatient care for small and sick newborns, our matrix detailed over 600 structural characteristics. Our review of the SPA, the SARA and the EmONC assessment tools identified several measurement omissions to capture information on key intervention areas, such as thermoregulation, feeding and respiratory support, treatment of specific complications (seizures, jaundice), and screening and follow up services, as well as specialised staff and service infrastructure.

**Conclusions:**

Our review delineates the required inputs to ensure readiness to provide inpatient care for small and sick newborns. Based on these findings, we detail where questions need to be added to existing tools and describe how measurement systems can be adapted to reflect small and sick newborns interventions. Such work can inform investments in health systems to end preventable newborn death and disability as part of the *Every Newborn* Action Plan.

The first 28 days of life, the newborn period, represents the time of highest risk in the human lifecycle. In 2016, an estimated 2.6 million newborns died [1], mainly of complications of prematurity (35%), infections (23%), and intrapartum complications leading to birth injury (24%) [1,2]. Preventing deaths from these causes requires a combined health systems approach [3] along the continuum of care. This approach should deliver routine newborn care for all babies (cleanliness, thermal care and support for breastfeeding), newborn resuscitation and prevention of mother to child transmission of HIV (PMTCT) for all babies who need it [4,5]; and timely provision of quality inpatient care for babies born small and sick [6,7].

Many low birth weight newborns, especially preterm infants, and those born small for gestational age, require support to feed and maintain their temperature. In addition, preterm newborns face increased risks of respiratory problems, infections and jaundice [8]. Even amongst those born at full term, significant numbers of newborns suffer from systemic infections, neonatal encephalopathy, pathological jaundice and congenital abnormalities, with high mortality risk in the absence of care [8]. “Small and sick newborns”, therefore, includes all those babies who require inpatient (facility-based) care to survive. The care that small and sick newborns require is not an individual intervention, but a package made up of multiple interventions. Previous work has discussed the specific evidence-based interventions that comprise this package of care [3,8,9], which are displayed in **Figure 1****.**

**Figure 1:**
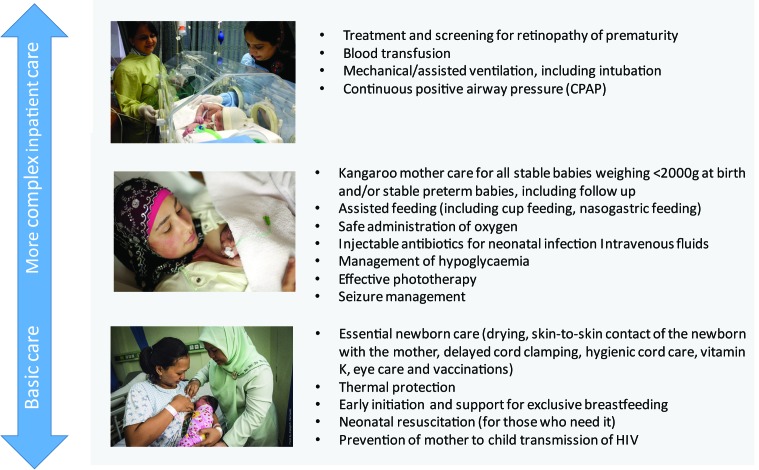
Evidence-based newborn care interventions from basic to complex care. There are additional evidence-based interventions for newborns that should be included in the antenatal period – antenatal corticosteroids and antibiotics for preterm premature rupture of membranes - and follow-up processes that would fall outside of the newborn period and be linked to paediatric services. Figure adapted from [8]. Photo credit (from top to bottom) Ayesha Vellani/Save the Children, ©EFCNI, JHPIEGO.

Delivery of interventions to small and sick newborns requires health facilities that are prepared, which is termed as “service readiness”. The underpinning principle to service readiness is based on traditional quality of care frameworks, such as that conceived by Donabedian (**Figure 2**). The framework refers to the structures (the necessary infrastructure, equipment, drugs, health providers and guidelines); and processes (actions performed by health professionals with requisite training and skills) that are needed to provide a package of care [8]. When all of the components of the structural domain are in place, it allows for improvements in clinical processes, which in turn lead to improvements in patient outcomes [10-12]. To achieve service readiness, the structures not only need to be present, but maintained, re-stocked and updated (eg, equipment requires maintenance, supplies require re-stocking, guidelines require updating) and staff continually trained and supervised. To deliver a quality package of care, therefore, requires strong health systems with the capacity to monitor and track service readiness and react appropriately to service needs.

**Figure 2:**
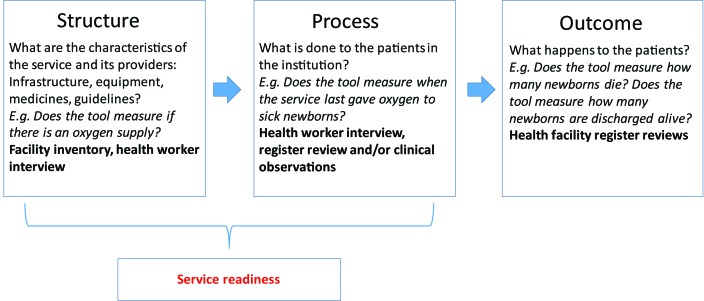
The Donabedian framework applied as a construct to map health facility assessment tools.

Evidence from *The Lancet* Every Newborn Series [13] informed the design of the *Every Newborn* Action Plan, a multi-partner initiative launched in 2014, backed by a World Health Assembly Resolution. *Every Newborn* aims to end preventable newborn deaths and stillbirths, with national targets of ≤12 neonatal deaths per 1000 live births and ≤12 stillbirths per 1000 total births by 2030 [7]. To achieve these targets, *Every Newborn* partners acknowledge a need to improve the measurement of care at birth, and to better track coverage, quality, and equity of care for small and sick newborns around the time of birth [14]. A dedicated sub-group – *Every Newborn* metrics - focuses on improving the measurement of interventions [14], and has a work stream focused on service readiness for inpatient care of small and sick newborns [9].

Currently, national and facility-based health information systems in low- and middle-income countries collect few data on service readiness for small and sick newborns [13-15], in contrast with child health programmes, notably immunisation, HIV and malaria [16]. Data are sparse in sub-Saharan Africa and parts of Asia where access to care for small and sick newborns is the lowest, and where many facilities need targeted efforts to strengthen services [8,14].

Data from functional routine national health management information systems (HMIS) and logistics management systems (LMIS) are able, in principle, to capture service readiness in a sustainable way, but the content and quality of data in national HMIS are variable in practice. This means many low- and middle-income countries depend on periodic evaluations, such as nationally representative facility surveys or censuses, as a key source of health information to monitor the readiness of the health system to provide facility-based care [14,17,18]. These surveys or censuses are referred to as health facility assessments.

The most common health facility assessment tools are the Demographic and Health Survey Programme’s Service Provision Assessment (SPA), the WHO Service Availability and Readiness Assessment (SARA) [19,20], and the EmONC assessments, currently managed by Averting Maternal Death and Disability (AMDD) [21] in collaboration with UNFPA [22]. The content of these tools with regards to service readiness specifically for inpatient care of small and sick newborns has not previously been systematically evaluated.

Our overall aim was to review the current health facility assessment tools’ ability to capture service readiness for inpatient care of small and sick newborns.

The specific objectives of this article are to:

1. Create a standardised matrix of the structural components (infrastructure, equipment, drugs, providers and guidelines) required to deliver inpatient care for small and sick newborns.

2. Compare the components of this standardised matrix against what is currently measured by widely used multi-country health facility survey tools (SPA, SARA, EmONC assessment) and identify gaps in measurement of the structural and process domains.

3. Synthesise these findings to provide recommendations on how to improve measurement of service readiness for inpatient care of small and sick newborns.

## METHODS

### Conceptual framework for service readiness

We applied the Donabedian framework [10,11] as a construct to map service readiness for small and sick newborns (**Figure 2**).

For the first objective, we mapped the structural domain of the framework and identified the infrastructure, equipment, drugs, health providers and guidelines required to deliver inpatient care for small and sick newborns. The second domain in the framework (process) links structures and outcomes, and is dealt with in objective 2.

### Objective 1: Create a standardised matrix of structural components 

#### Development of matrix of service readiness requirements for inpatient care of small and sick newborns

We listed a total of 17 newborn interventions based on work done previously as part of the *Every Newborn* metrics process [5,8,23] (**Figure 1**). All newborn interventions were included for this exercise, including essential newborn care, based on the rationale that small and sick newborns will require these basic interventions in addition to inpatient care [8,9].

We organised the matrix by six areas: 1) labour and delivery room 2) place of care for small and sick newborns 3) pharmacy/medicines, 4) human resources/providers, 5) laboratory & blood bank and 6) referral service. We organised interventions by whether they occur in the labour and delivery room or the neonatal unit (or both). Human resources and pharmacy were allocated as a separate area given that providers and drugs will be needed in multiple places of care. Given that most of the guidelines reviewed included information or guidance on referral systems and the associated structural components, we included referral system as a separate “area”. Finally, given the wide variation in laboratory systems, we separated the laboratory and blood bank by capacity to perform certain tests and actions, rather than an itemised list of components or equipment.

We searched for existing published guidelines for all the newborn interventions, including relevant newborn and paediatric guidelines available on the WHO website. Where no WHO guideline existed, we consulted relevant resources developed by UNICEF and other partners, including resources from international professional associations, such as the American Academy of Paediatrics and Royal College of Paediatrics and Child Health. To ensure consistency with other areas of care, we also reviewed the interagency list of medical devices for essential interventions for reproductive, maternal, newborn, and child health [24], a master list created for newborn health in humanitarian settings by the Inter-Agency Working Group on Reproductive Health in Crises (IWAG), UNICEF and Save the Children [25] and the latest version of the WHO model essential drugs list [26]. See **Table 1** for a list of the guidelines and resources used for this review.

**Table 1:** Resource materials and guidelines reviewed for newborn interventions

Intervention	Resource material or guideline	Year published
Essential newborn care, thermal protection, early initiation and support for exclusive breastfeeding	WHO essential newborn care course	2010
	WHO early essential newborn care: Clinical practice pocket guide	2014
	Essential care for every baby	2015
	WHO Integrated management of pregnancy and childbirth: Pregnancy, Childbirth, Postpartum and Newborn Care: A guide for essential practice	2015
	UNICEF: Baby Friendly Hospital Initiative (BFHI)	2012
	WHO Interagency list of priority medical devices for essential interventions for reproductive, maternal, newborn and child health	2015
Neonatal resuscitation	WHO guidelines on basic newborn resuscitation	2012
	Helping Babies Breathe Resources	2017
	WHO guidelines on managing complications in pregnancy and childbirth	2007
	WHO guidelines on managing newborn problems: a guide for doctors, nurses and midwives	2003
Prevention of mother to child transmission of HIV	WHO guideline update on HIV and infant feeding	2016
	WHO guidelines on antiretroviral drugs for treating pregnant women and preventing HIV infection in infants	2010
	Médecins sans Frontières: Neonatal Care Guidelines	2016
Kangaroo mother care for premature babies, including follow up, alternative feeding (cup feeding and nasogastric feeding)	Essential care for small babies	2015
	WHO kangaroo mother care: A practical guide	2003
	WHO guidelines on optimal feeding of low birth-weight infants in low- and middle-income countries	2011
	UNHCR operational guidelines on improving newborn health in refugee operations	2014
	UNICEF toolkit for setting up special care newborn units, stabilisation units and newborn care corners	2015
	WHO recommendations on interventions to improve preterm birth outcomes	
Injectable antibiotics for neonatal infections, hypoglycaemia management, effective phototherapy, seizure management, administration of oxygen	WHO pocket book of hospital care for children	2013
	Save the Children, UNICEF: Newborn care charts	2009
Treatment and screening for retinopathy of prematurity*	Guidelines on screening and treatment for retinopathy of prematurity (UK and India)	2008
Blood transfusion, Mechanical ventilation and continuous positive airway pressure (CPAP)	WHO pocket book of hospital care for children	2013

*No current WHO guidelines available; recent guidelines recommended by ROP experts from India and UK selected for review.

Within each area of the matrix, where equipment items recurred (eg, components required for more than one intervention such as linen, gauze, swabs, weighing scale) we included these under general items for either the labour and delivery room or place of care for small and sick newborns. We were then able to populate the matrix with the specific components required to deliver the interventions for small and sick newborns.

### Objective 2: Compare the components of this standardised matrix against widely used multi-country health facility survey tools (SPA, SARA, EmONC assessment) and identify gaps in measurement of structural and process domains

We obtained the latest versions of the SARA (version 2.2, revision July 2015) [20] and the SPA (revised 2012) [27] from their websites. The EmONC assessment tool was being revised at the time of the study and we obtained the version undergoing field-testing from AMDD in July 2016.

We reviewed the SARA core questionnaire tool, the SPA health facility inventory and health worker interview, and the latest versions of the relevant modules from the EmONC assessment, (Module 1: Identification of facility and infrastructure; Module 2: Human Resources; Module 3: Essential drugs, equipment and supplies; Module 5: EmONC interventions; and Module 7: Provider knowledge and competency for maternal & newborn care).

We compared the content of each of the tools to the structural components in our matrix. To identify gaps in structural components we checked:

- Does the tool measure the infrastructure, equipment, drugs, health providers and/or guidelines needed to provide the interventions?

Many of the tools are designed to also measure aspects of the process domain in the service readiness framework (**Figure 2**). Therefore, measurement of regular practice or training was considered as a proxy measurement of the process domain for service readiness (as it looks at what is regularly done to patients in the institution). For each of the 17 interventions included in the package of care (**Figure 1**), we also checked:

- Does the tool measure whether staff are given any training to provide the intervention?
- Does the tool measure if the intervention is regularly performed?

The first author (SM), conceptualised the matrix and completed the review of each tool. The matrix was then verified for completeness by practicing neonatologists and nurses with experience in neonatal care in Ghana, Nigeria and co-author practicing neonatal clinicians with experience in India, Malawi, Uganda, United Kingdom and United States.

The review of the health facility assessment tools was verified by a representative of the lead agency for the EmONC assessment and the SPA to ensure the findings were consistent with the most recent versions of the tool.

## RESULTS

### Objective 1: Create a standardised matrix of structural components 

We mapped a total of 654 service readiness items for inpatient care of small and sick newborns to provide 17 interventions. This included a total 167 structural items in the labour and delivery room and 266 in the place for small and sick newborn care (or neonatal unit). We listed a total of 33 different potential providers, 114 essential newborn drugs and medicines. A summary list of the international resource materials and guidelines available for care of small and sick newborns used for this objective is included in **Table 1**. The essential drug list is shown in **Table 2**. The complete matrix is available in the supplementary material (Table S1 in **Online Supplementary Document(Online Supplementary Document)**).

**Table 2:** Example minimum drug list for inpatient care of small and sick newborns showing rationale for use in newborns and summary of the Service Provision Assessment (SPA), Service Availability and Readiness Assessment (SARA) and Emergency Obstetric and Newborn Care (EmONC) Assessment tools and Essential Medicines List*

Drug name	SPA	SARA	EmONC	EML	Drug description/use
**Antibiotics:**					
Amoxicillin (oral suspension)	Y	Y	Y	Y	Penicillin antibacterial for neonatal infections
Amoxicillin (injection)			Y	Y	Penicillin antibacterial for serious neonatal infections
Amikacin (IV or IM)				Y	Aminoglycoside antibacterial; alternative treatment of opthalmia neonatorum
Ampicillin (IV or IM)	Y	Y	Y	Y	Penicillin antibacterial for serious neonatal infections
Ampicillin (oral)					Penicillin antibacterial for neonatal infections
Azithromycin (oral)	Y	Y		Y	Macrolide antibacterial for P-PROM (maternal use)
Benzathine benzylpenicillin (benzathine penicillin G) (IM)	Y	Y		Y	Penicillin antibacterial for treatment of congenital syphilis
Benzylpenicillin (Penicillin G) (IV or IM)	Y		Y	Y	Penicillin antibacterial for serious neonatal infections
Cefalexin (oral suspension)				Y	First generation cephalosporin used in newborns for skin and soft tissue infections
Cefotaxime (IV or IM)			Y	Y	First generation cephalosporin with broad spectrum for treatment of serious neonatal infections
Ceftriaxone (IV or IM)	Y	Y	Y		Third generation cephalosporin for neonatal infections, genital gonococcal and/or chlamydial infection
Ciprofloxacin (injection)				Y	Second generation fluoroquinolone antibacterial sometimes used as second line treatment
Ciprofloxacin (oral)	Y	Y		Y	Second generation fluoroquinolone antibacterial for treatment of bacterial diarrhoea
Clindamycin (IV)			Y	Y	Lincosamide antibacterial, second line treatment (eg, streptococcal or soft tissue infections)
Co-amoxiclav (oral suspension)			Y		Penicillin antibacterial, can be used where no IV access
Co-amoxiclav (injection)					Penicillin antibacterial used for neonatal skin infections
Cotrimoxazole (oral)	Y	Y			Combined antibacterial for prophylactic treatment of HIV
Erythromycin (oral)	Y		Y	Y	Macrolide antibacterial for P-PROM (maternal use)
Flucloxacillin (IV/IM) (cloxacillin)			Y	Y	Penicillin antibacterial treatment for neonatal sepsis
Flucloxacillin (oral)			Y	Y	Penicillin antibacterial. Can be used in newborns as follow on from intravenous flucloxacillin
Gentamicin (IM or IV)	Y	Y	Y	Y	Aminoglycoside antibacterial used for treatment of neonatal sepsis
Isoniazid (oral)	Y	Y		Y	Antituberculous antibacterial used occasionally for congenital TB
Kanamycin				Y	Aminoglycoside antibacterial; alternative to gentamicin
Metronidazole (IV)	Y	Y	Y	Y	Antiprotozoal antibacterial used for neonatal meningitis and/or anaerobic bacterial infections
Metronidazole (oral)	Y	Y		Y	Antiprotozoal antibacterial used for neonatal meningitis and/or anaerobic bacterial infections
Procaine benzylpenicillin (IM)		Y	Y	Y	Penicillin antibacterial used for congenital syphilis
Tetracycline 1% eye ointment	Y	Y	Y	Y	Prophylactic topical antibiotic used to prevent bacterial (eg, chlamydial, gonococcal) neonatal conjunctivitis
**Anticonvulsants:**					
Diazepam (oral/NG)				Y	Sedative, anticonvulsant, muscle relaxant, mostly used for neonatal tetanus
Diazepam emulsion (IV)	Y	Y	Y	Y	Sedative, anticonvulsant, muscle relaxant used for neonatal tetanus
Midazolam (oral solution)				Y	Sedative, anticonvulsant used for seizures
Paraldehyde (rectal)					Anticonvulsant for seizures
Phenobarbital (IV or IM)		Y	Y	Y	First line anticonvulsant for tonic clonic and partial seizures
Phenobarbital (oral)		Y		Y	First line anticonvulsant for tonic clonic and partial seizures
Phenytoin (IV)			Y	Y	Anticonvulsant for tonic clonic and partial seizures
**Emergency drugs:**					
Adrenaline/epinephrine (IV)	Y	Y	Y	Y	Sympathomimetic for cardiopulmonary arrest used for advanced neonatal resuscitation
Aminophylline			Y		Methylzanthine used to prevent apnoeic attacks in premature newborns
Atropine (injection)		Y	Y	Y	Parasympatholytic, antispasmodic used for intubation
Calcium gluconate (injection)	Y	Y	Y	Y	Used for hypocalcaemic seizures and hyperkalaemia
Hydrocortisone (injection)	Y		Y	Y	Steroidal anti-inflammatory used for hypotension or severe broncho-pulmonary dysplasia
Magnesium sulphate (IV)	Y	Y	Y	Y	Inorganic salt compound, maternal use in preterm labour, protective against cerebral palsy
Naloxone (IV)			Y	Y	Specific opioid antagonist for respiratory depression in newborns
**Analgesics:**					
Ibuprofen (IV)				Y	Analgesic sometimes used in newborns for closing patent ductus arteriosus
Morphine (IV)	Y	Y		Y	Centrally acting opioid analgesic for severe pain, sedation and intubation
Morphine (oral)		Y	Y	Y	Used for severe pain
Paracetamol (oral)	Y	Y	Y	Y	Analgesic for minor pain
Paracetamol (suppository)				Y	Analgesic for minor pain
Paracetamol (injection)					Analgesic for minor pain. Also used for newborns for closing patent ductus arteriosus.
**Corticosteroids:**					
Betamethasone (IM)	Y	Y	Y		Not used in newborns; used in mothers with threatened preterm labour <34 weeks gestation for fetal lung maturation
Dexamethasone (IM)	Y	Y	Y	Y	Not used in newborns; used in mothers with threatened preterm labour <34 weeks gestation for fetal lung maturation
**IV fluids:**					
Calcium gluconate 10%	Y	Y		Y	Supplement used to treat calcium deficiency. Dependent on programme context – careful monitoring required
Dextrose 10% with normal saline	Y		Y	Y	Solution used for maintainance fluid therapy
Dextrose/glucose 5%	Y	Y	Y	Y	Solution used as vehicle for administration of IV drugs
Dextrose/glucose 10%		Y	Y	Y	Solution for treatment of hypoglycaemia and maintenance fluid therapy on first day of life for sick babies who cannot feed
Potassium chloride (KCl) 7.5%, 10%, 15%				Y	Solution only to be used in contexts where monitoring of potassium levels is available.
Sodium bicarbonate				Y	Solution used to dissolve artesunate
Sodium chloride 0.9%	Y	Y	Y	Y	Solution used as a vehicle for administration of IV/parenteral drugs, fluid replacement and flushing IV lines
Ringer’s lactate	Y	Y	Y	Y	Compound solution for severe dehydration/hypovolaemia can be added to dextrose/glucose for a mix
Water for injection					Sterile water for mixing drugs
**Anti-malarials:**					
Artesunate (IV or IM)	Y	Y		Y	First line treatment for neonatal malaria
Artesunate (rectal)	Y	Y		Y	Neonatal malaria treatment if IV/IM access not available
Arthemeter (IM)				Y	Second line treatment for neonatal malaria
Artemisinin-based combined therapy (oral)	Y	Y	Y	Y	Second line anti-malarial treatment followed by ACT
**Antiretrovirals (may vary depending on national HIV guidelines):**					
Azidothymidine/Zidovudine (AZT) (oral)	Y	Y	Y	Y	Antiretroviral
Lamivudine	Y	Y	Y	Y	Antiretroviral
Nevirapine (NVP) (oral)	Y	Y	Y	Y	Antiretroviral
**Other drugs:**					
Aciclovir (IV)				Y	Antiviral used for herpes
Acyclovir 3% topical eye ointment				Y	Antiviral active against herpes virus used to prevent neonatal herpes keratitis in babies born to mother with genital herpes
Anti-Rho (D) immune globulin (injection) *			Y		To prevent Rhesus disease (haemolytic disease of the newborn) given to mothers
Caffeine citrate (oral)				Y	Preventive treatment for apnoea
Caffeine citrate (IV)				Y	Preventive treatment for apnoea, oral preferred over IV
Chlorhexidine digluconate 7.1% gel (delivering 4% chlorhexidine)	Y		Y	Y	Topical treatment of omphalitis
Domperidone					Anti-reflux drug for gastro-oesophageal reflux
Ethambutamol (oral)	Y	Y		Y	First line oral anti-tuberculolous drug
Ferrous fumerate (oral)	Y		Y	Y	Oral suspension used for preterm neonates to prevent iron deficiency
Folic acid	Y	Y	Y	Y	Oral suspension used for folate supplementation
Fluconazole (IV)				Y	Antifungal drug used in newborns over 1 week
Fluconazole (oral)	Y	Y	Y	Y	Antifungal drug
Furosemide (IV)		Y		Y	Diuretic used for chronic lung disease, oedema in advanced settings
Furosemide (oral)	Y		Y	Y	Diuretic
Glycerin chip					Suppository used in newborns to stimulate stooling
Hepatitis B immune globulin (HBIG)					Treatment of Hepatitis B in neonates
Human milk fortifier					Fortifier, adds protein, calories and micronutrients to expressed breastmilk for LBW babies
Insecticxide treated bed nets (in malaria endemic areas)	Y	Y	Y		For mother’s beds in KMC ward and for discharge home
Lidocaine solution	Y	Y	Y	Y	Local anaesthetic
Miconazole cream (or equivalent eg, gentian violet)	Y			Y	Topical antifungal for candida dermatitis used for nappy area
Multivitamin					Containing zinc, vitamin A etc.
Nystatin (oral solution)	Y		Y	Y	Topical antifungal for oropharyngeal candidiasis used prophylactically with antibiotic treatment
Nystatin cream				Y	Topical antifungal
Omeprazole (IV)	Y			Y	Acid blocker for gastro-oesophageal reflux
Omeprazole (oral)	Y			Y	Acid blocker for gastro-oesophageal reflux
Oral rehydration solution	Y	Y	Y	Y	Powder to mix with drinking water for oral rehydration; breastmilk feeding should be encouraged
Oxygen supply				Y	Medical inhalation gas for treatment of respiratory distress
Phosphate and calcium supplements					Supplementation
Potassium Chloride (1mmol/ml) (oral)				Y	Powder solution for maintainance oral potassium replacement
Pyridoxine (oral)				Y	Preventive therapy for tuberculosis
Pyrazinamide (oral)	Y	Y		Y	First line oral anti-tuberculolous drug
Ranitidine (IV)				Y	Antacid drug for gastro-oesophageal reflux
Ranitidine (oral)				Y	Antacid drug for gastro-oesophageal reflux
Rifampicin (oral)	Y	Y		Y	First line oral anti-tuberculolous drug
Sucrose 30% (oral)					Non-pharmacological pain management for minor procedures (eg, cannulation)
Tetanus immunoglobulin (HTIG) (IM)			Y	Y	Anti-tetanus immunoglobulin for treatment of neonatal tetanus
Vitamin B6 (pyridoxine) (IV or IM)					Vitamin for B6 deficiency
Vitamin D					Supplementation.
Vitamin K1 (Phytomenadione) (IM or IV)			Y	Y	Vitamin and anti-haemorrhagic for prophylactic treatment of haemorrhagic disease of the newborn
Water based lubricant					For inserting suppositories and/or other procedures.
Zinc oxide cream					Topical for nappy/diaper rash
Vaccines:					
BCG vaccine	Y	Y	Y	Y	Prevention of TB
Diptheria	Y	Y		Y	Prevention of diptheria
Pertussis vaccine	Y	Y		Y	Prevention of pertussis
*Haemophilus influenzae* type b (Hib) vaccine	Y	Y		Y	Prevention of haemophilis influenzae type B
Hepatitis B vaccine	Y	Y		Y	Prevention of hepatitis B in countries where perinatal infection is common, as per vaccination schedule
Oral poliomyelitis vaccine	Y	Y	Y	Y	Prevention of poliomyelitis
Tetanus toxoid	Y	Y	Y	Y	Prevention of tetanus in wound management, prevention of maternal and neonatal tetanus in pregnant women

EmONC – Emergency Obstetric and Newborn Care, SPA – Service Provision Assessment, SARA – Service Availability and Readiness Assessment, EML – Essential Medicines List, IM – intramuscular, IV – intravenous, NG – nasogastric

*Y – measured by the tool.

### Objective 2: Compare the components of this standardised matrix against widely used multi-country health facility survey tools (SPA, SARA, EmONC assessment) and identify gaps in measurement of structural and process domains

The SPA, the SARA and the EmONC assessment tools are summarised in **Table 3**. All three tools have different purposes, are measured at different intervals, and have different approaches to measurement and sampling.

**Table 3:** Summary of three multi-country health facility assessment tools: Service Provision Assessment (SPA), Service Availability and Readiness Assessment (SARA) and Emergency Obstetric and Newborn Care (EmONC) Assessment

	Service Provision Assessments (SPA)	Service Availability and Readiness Assessment (SARA)	Emergency Obstetric and Newborn Care (EmONC) Assessment
**Purpose of tool**	For comprehensive monitoring of a country’s formal health care system; monitors the overall availability of different facility-based health services in a country and their readiness to provide those services	For assessing readiness of facilities using a standard set of indicators that cover all main health programmes. Only designed to assess service readiness (not performance or client perspectives)	For monitoring and assessment of the availability, use and quality of routine and emergency obstetric and newborn care in the formal health system.
**Organisation(s)**	The Demographic and Health Survey (DHS) Program, United States Agency for International Development (USAID)	World Health Organization (WHO), USAID	Averting Maternal Death & Disability (AMDD), United Nations Population Fund (UNFPA), United Nations Children’s Fund (UNICEF), WHO.
**Sample**	Sample survey or census of formal sector health facilities designed to provide nationally representative results by facility type, managing authority, and geographic region.	Sample survey or census of at least 150 public and private facilities	Census of hospitals and census or sample of lower-level delivery sites (public and private facilities). Sample may be random or selection may be restricted to lower-level facilities that meet a specific volume of deliveries.
**Modules**	Facility inventory, exit interviews (antenatal care, family planning, sick child), clinical observations (antenatal care, family planning, sick child), health worker provider interviews	Facility inventory, health worker interview	Facility inventory, human resources, essential drugs, equipment and supplies, facility case summary, Emergency Obstetric Care (EmOC) signal functions, provider knowledge for maternal and some newborn care & chart reviews.
**Numerator for indicator**	Number of facilities ready to provide MNCH, family planning, HIV/AIDS, STIs, Malaria, Tuberculosis, basic surgery, non-communicable diseases services.	Proportion of health facilities, number of core medical professionals, proportion of facilities offering a defined service and the density and distribution of the facilities	Number of facilities providing EmOC, number of facilities providing each EmOC signal functions by level of care.
Denominator for indicator*	All formal facilities	All facilities, per 10 000 population	All surveyed facilities by level of care; availability of EmOC is measured per 500 000 population or 20 000 births*
**Timeframe**	15-18 months to complete fieldwork and report	Variable, but shorter than SPA or EmONC	12-18 months to complete field work and report
**Frequency**	4-5 yearly intervals	Designed to be repeated annually	4-5 yearly intervals

*Discussion is ongoing on whether denominator should measure births or population); expected number of births is the denominator for several other indicators – institutional birth rate, caesarean-section rate, met need for emergency obstetric care.

**Table 4** and **Table 5** summarise the mapping of the interventions showing the structures and processes currently measured by the SPA, the SARA and the EmONC survey tools, and highlighting gaps in measurement of structural and process domains.

**Table 4:** A summary of the Service Provision Assessment (SPA), Service Availability and Readiness Assessment (SARA) and Emergency Obstetric and Newborn Care (EmONC) assessment tools’ capacity to measure structural and process domains of service readiness for newborn interventions in the labour and delivery room

Intervention and components of structural domain	Health facility assessment tool		
	**SPA**	**SARA**	**EmONC**
**Immediate/essential newborn care:**			
Infrastructure	Y	Y	Y
Equipment & drugs	Y	Y	Y
Guidelines		Y	Y
Training	Y	Y	Y
Routine practice	Y	Y	Y
**Thermal protection:**			
Infrastructure	Y	Y	Y
Equipment & drugs			
Guidelines	Y	Y	Y
Training	Y		
Routine practice	Y		Y
**Immediate and exclusive breastfeeding:**			
Infrastructure			
Equipment & drugs			Y
Guidelines	Y		Y
Training	Y	Y	Y
Routine practice	Y	Y	Y
**Resuscitation with bag and mask:**			
Infrastructure	Y	Y	Y
Equipment & drugs	Y	Y	Y
Guidelines	Y	Y	Y
Training	Y	Y	Y
Routine practice	Y	Y	Y
**PMTCT if HIV-positive mother:†**			
Infrastructure	Y	Y	Y
Equipment & drugs	Y	Y	Y
Guidelines	Y	Y	Y
Training	Y	Y	Y
Routine practice	Y	Y	Y

EmONC – Emergency Obstetric and Newborn Care, SPA – Service Provision Assessment, SARA – Service Availability and Readiness Assessment

*Y – measured by the tool.

†May only be applicable in settings with high HIV prevalence.

**Table 5:** A summary of the Service Provision Assessment (SPA), Service Availability and Readiness Assessment (SARA) and Emergency Obstetric and Newborn Care (EmONC) tools’ capacity to measure the structural and process domain of service readiness for interventions in the newborn inpatient care unit

Intervention and components of structural domain	Health facility assessment tool		
	**SPA**	**SARA**	**EmONC**
**Kangaroo mother care (KMC) including follow up:**			
Infrastructure			
Equipment & drugs			
Guidelines			Y
Training	Y		Y
Routine practice	Y	Y	Y
**Alternative feeding if baby unable to breastfeed (cup feeding and nasogastric feeding):**			
Infrastructure			
Equipment & drugs			
Guidelines			
Training			
Routine practice			Y
**Safe administration of oxygen (including equipment for resuscitation):**			
Infrastructure	Y	Y	Y
Equipment & drugs			Y
Guidelines			Y
Training			Y
Routine practice			Y
**Intravenous fluids and management of hypoglycaemia:**			
Infrastructure			
Equipment & drugs			Y
Guidelines			Y
Training	Y		Y
Routine practice		Y	Y
**Injectable antibiotics for neonatal infection:**			
Infrastructure	Y	Y	Y
Equipment & drugs	Y	Y	Y
Guidelines	Y	Y	Y
Training	Y		Y
Routine practice		Y	Y
**Effective phototherapy:**			
Infrastructure			
Equipment & drugs			
Guidelines			
Training			
Routine practice			
**Seizure management:**			
Infrastructure	Y	Y	Y
Equipment & drugs	Y	Y	Y
Guidelines			
Training			
Routine practice			
**Continuous positive airway pressure and assisted/mechanical ventilation:**			
Infrastructure	Y	Y	Y
Equipment & drugs			
Guidelines			
Training			
Routine practice			
**Blood transfusion for newborns:**			
Infrastructure	Y	Y	Y
Equipment & drugs			
Guidelines			
Training			
Routine practice			
**Treatment and screening for retinopathy of prematurity:**			
Infrastructure			
Equipment & drugs			
Guidelines			
Training			
Routine practice			

EmONC – Emergency Obstetric and Newborn Care, SPA – Service Provision Assessment, SARA – Service Availability and Readiness Assessment

*Y – measured by the tool.

For ease of presentation, and to avoid repetition, we summarise the findings from the review in this section by structural and process domains.

### Comparison of the matrix against health facility assessment tools and identification of gaps in measurement of the structural domain

#### Infrastructure

All three tools measured elements of general health facility infrastructure, such as electricity supply, means of communication, referral and transport and availability of water, toilets/latrines and waste disposal.

All tools measure availability of a table or surface for performing resuscitation. However, infrastructural requirements to support essential newborn care both in the labour and delivery room and the postnatal ward, such as space, privacy (screens) for mother to express breastmilk and infrastructure for storage of breastmilk (and whether there is consistent power supply for refrigeration) were not measured by any of the tools. All tools collect details on infrastructure to provide PMTCT.

The SPA measured space for mothers to provide kangaroo mother care (KMC) in its facility inventory, but only the EmONC assessment asked about space allocation for sick newborn care or a special care unit (eg, infrastructure to provide services beyond KMC, such as assisted feeding, thermal protection, fluids and/or oxygen support). As oxygen is a crosscutting infrastructural component needed for several interventions outside of newborn health, all tools measured availability of an oxygen source. However, none of the tools measured the newborn-specific infrastructure that would be needed for safe oxygen therapy. Continuity of electricity and oxygen is especially important for facilities offering care for small and sick newborns who may be dependent on consistent oxygen source and/or electric equipment. None of the tools measured service readiness infrastructure for screening services (for example, developmental milestones, hearing and vision) or follow-up for high-risk infants.

None of the tools measured advanced infrastructure for intensive care for very small and sick newborns, such as that required for mechanical ventilation, newborn blood and/or exchange transfusion, and specialist laboratory infrastructure beyond that needed for obstetric, and some paediatric and adult services.

#### Equipment

All tools measured provision of basic equipment for neonatal resuscitation, including smaller-sized face masks and resuscitation bag in the labour and delivery room. None of the tools measured whether resuscitation equipment was available in the room where small and sick newborns are cared for to ensure safety and continuity of care.

Simpler interventions for small newborns, such as assisted feeding (plastic feeding cups and small sized nasogastric tubes) were only measured by the EmONC assessment, and hats or caps (including small sizes) are not consistently measured among the tools.

Phototherapy equipment needed to treat neonatal jaundice was only measured by the EmONC assessment (fluorescent tubes and icterometry). Lower cost phototherapy technologies, such as LED phototherapy devices were not included in any of the tools.

Although the infrastructure for oxygen was measured, most likely for paediatric and adult services, safe delivery of oxygen to newborns requires significant additional equipment items, such as newborn pulse oximetry, neonatal nasal prongs, oxygen-air blenders, low-flow metres and humidifiers, which were not captured by the tools.

Higher level respiratory support for newborns, such as Continuous Positive Airway Pressure (CPAP) ventilation, was not measured by any of the tools. Our matrix shows that for safe delivery of CPAP, beyond the drivers themselves, facilities would require critical emergency equipment in case of pneumothorax such as transilluminators, chest tubes and valves.

Intubation equipment (eg, laryngoscopes blades in small sizes) were measured by the EmONC assessment, but other critical components to support a ventilated newborn were not measured, including the ventilator machine.

#### Drugs

The EmONC assessment tool had the most extensive list of drugs and medicines for newborns detailing 106 medicines and drugs for mothers and newborns, but very few of these are specified for newborns. There were several notable omissions of medicines for care at birth within the SPA and SARA, such as vitamin K (SPA only asks whether it is routinely administered).

All three tools measured antibiotic drugs for treating small and sick newborn infections (amoxicillin oral and injection, ampicillin injection and gentamicin injection as a minimum). However, inventories did not specify whether the antibiotic was available in the injectable form, with the appropriate concentrations and diluents (usually water for injection, sodium chloride 0.9% and glucose 5%), or the availability of smaller intravenous cannulas/catheters and syringe drivers. The tools measured standard intravenous fluid preparations, but only EmONC included glucose 10%, which is most frequently used for neonates. For seizure management, only the EmONC tool measured the first and second line treatments (intravenous phenobarbitone and phenytoin).

Several drugs that might be used for advanced level care, such as procedural sedation and pain relief, were not currently included in any of the tools.

In **Table 2** we present an example drug list for inpatient care of small and sick newborns indicating which drugs are measured by each tool and whether these are on the most recent WHO model essential medicines list [26]. This includes the commodities needed for retinopathy of prematurity screening and treatment, such as dilating and anaesthetic eye drops, which are not currently included in existing tools.

#### Health providers

There were several notable gaps in measurement of specialist newborn staff. Only the EmONC tool measured specialist staff cadres for newborns (eg, neonatologist) and none of the tools measured specialist neonatal nurses. Allied staff and support staff (eg, social workers, speech therapist) were not measured. None of the tools measured ophthalmologists or related professions that are needed in settings where newborns may require screening and treatment for retinopathy of prematurity, or biomedical engineers for equipment maintenance.

#### Guidelines

**Table 1**. shows all the guidelines and educational resources used for this review. Some resources cover a number of different interventions. However, there were notable gaps in available guidelines for more complex interventions, such as continuous positive airway pressure, blood transfusion, exchange transfusion, ventilation, and treatment and screening of retinopathy of prematurity.

### Identification of gaps in measurement of the process domain to capture service readiness to care for small and sick newborns for each intervention

Measurement of regular practice or training of health staff in specific interventions was considered as a proxy measurement for the process domain for service readiness as it looks at what is regularly done to patients in the institution.

#### Regular practice

All the tools relied on direct health worker reports, the register, or chart reviews to measure whether select interventions were regularly provided for small and sick newborns.

The SPA looked at whether a limited number of interventions relevant to newborns (neonatal resuscitation and corticosteroids for preterm labour) were ever practiced and practiced in the last 3 months. The SPA also included a series of questions on essential care for newborns, but none on inpatient care for small and sick newborns, other than if KMC was practiced in the facility.

The SARA asked whether a limited number of functions were provided in the last 12 months: antibiotics for preterm or prolonged premature rupture of membranes, antenatal corticosteroids, neonatal resuscitation, KMC, and injectable antibiotics.

The EmONC assessment had the most detailed list of newborn interventions for which questions on regular or recent practice were asked (newborn resuscitation, antenatal corticosteroids, antibiotics for preterm premature rupture of membranes, antibiotics for neonatal infections, KMC, administration of oxygen and administration of IV fluids).

The EmONC tool included specific knowledge questions on small and sick newborn care, including a few interventions, such as resuscitation, oxygen therapy and infections.

**Table 6** summarises the approaches used by each of the tools to capture regular practice and training.

**Table 6:** The approach used by Service Provision Assessment (SPA), Service Availability and Readiness Assessment (SARA) and Emergency Obstetric and Newborn Care (EmONC) to measure regular practice and training

	SPA		SARA		EmONC	
**Regular practice**	**Training**	**Regular practice**	**Training**	**Regular practice**	**Training**
Essential newborn care	Routinely practiced	Training in last 24 months	Routinely carried out	Training in last 24 months	Performed in last 3 months	Ever received training
Thermal protection		Training in last 24 months			As part of essential newborn care	As part of essential newborn care
Early initiation and support for exclusive breastfeeding	Routinely practiced	Training in last 24 months	Routinely carried out	Training in last 24 months	As part of essential newborn care	As part of essential newborn care
Neonatal resuscitation with bag and mask	Ever practiced, practiced in last 3 months	Training in last 24 months	Practiced in last 12 months	Training in last 24 months	Performed in last 3 months	Ever received training
Prevention of mother to child transmission of HIV	Routinely practiced	Training in last 24 months	Service is offered	Training in last 24 months	ARVs given to newborns in the last 3 months	Ever received training
Kangaroo mother care	Ever practiced	Training in last 24 months	Practiced in last 12 months		Performed in the last 3 months	Ever received training
Assisted feeding (cup feeding and nasogastric feeding)					Performed in last 3 months	
Safe administration of oxygen					Performed in last 3 months	
Injectable antibiotics for neonatal infection		Training in last 24 months	Practiced in last 12 months		Performed in last 3 months	Ever received training
Intravenous fluid		Training in last 24 months			Performed in last 3 months	

EmONC – Emergency Obstetric and Newborn Care, SPA – Service Provision Assessment, SARA – Service Availability and Readiness Assessment

The synthesis of these findings to provide recommendations on improving these measurements is provided in the Discussion section.

## DISCUSSION

We have mapped the service readiness requirements for inpatient newborn care, detailing a total of 654 structural components to deliver 17 newborn interventions. Our review of three health facility assessment tools identified measurement gaps for almost all newborn interventions, even for the more basic interventions, such as thermoregulation and feeding. The most significant measurement gaps are for more complex interventions, which are currently not captured by any of the tools in our review. We found many commonalities among these tools, but also highlighted important differences that show how they have evolved with important, but distinct purposes, and different measurement approaches [9]. The size and cost of these assessments already limits the frequency of carrying out these surveys; adding a long list of indicators for small and sick newborn care would compound this challenge [28]. To improve the existing tools, we found that a number of indicators for basic service readiness could be harmonised, and some proxy indicators of service readiness for more complex care could potentially be added. As with other more complex areas of care, monitoring all the structures and processes for small and sick newborns will likely require a facility-based monitoring system [29-31].

The existing, up-to-date care guidelines for inpatient care for small and sick newborns are mainly split between obstetric care and paediatric care (**Table 2**) [32-35]. To the best of our knowledge, this is the first time that service readiness for small and sick newborn care has been delineated and mapped by structural component. The resulting matrix can be used by implementers for programme planning, depending on the needs of their health system and the interventions or packages of care they intend to provide at their service (Table S1 in **Online Supplementary Document(Online Supplementary Document)**). It is a step towards developing a more general facility based monitoring system or core module. Following validation, such a tool could be adapted for different settings as has been done in India [29].

The following sections provide a synthesis of findings and recommendations for improving the widely used tools for measurement of small and sick newborn care.

### Harmonisation of existing health facility assessment tools

#### Indicators

The interventions that are best represented by the existing tools are those that have been promoted as vertical programmes, such as neonatal resuscitation (which is a core indicator for obstetric and newborn care assessments), essential newborn care (for all babies) and PMTCT. The measurement approach and indicators for many of the more basic newborn interventions would benefit from more standardisation between tools. As a minimum, this should include service readiness indicators for essential newborn care (including service readiness for drying, skin-to-skin contact, cord clamping, vitamin K and initiation of breastfeeding), neonatal resuscitation and kangaroo mother care [28].

All of the existing tools have some questions on KMC, but for monitoring of operational KMC [28], the facility inventories require adaptations to incorporate more of the items needed for KMC [23] including the equipment for feeding support, antibiotics and amenities for mothers to stay in the facility [14]. Whilst listing the items needed for antenatal care exceeded the scope of this exercise, these should be considered in future tools, such as availability of antenatal corticosteroids for threatened preterm labour and antibiotics for preterm rupture of membranes (per WHO guidelines) as a minimum.

Measurement of training and skills for newborn interventions could be harmonised between tools such that these indicators are comparable between different surveys (see **Table 4**).

### Crosscutting service readiness needs

Health providers, especially midwives and specialist nurses, play a critical role in neonatal care [6,8,36-38]. Specially trained neonatal nurses may not be available in all health facilities, but previous studies show it is important to monitor who, if anyone, cares for newborns in the absence of specialised staff [8]. Recent studies in higher income settings, where neonatal nursing is a specialist cadre, show that reducing the nurse-to-patient ratio in neonatal units increases in-hospital mortality [39,40]. As a minimum, all health facility assessment survey tools could include questions on staff rotation policies to ensure specialist staff are not regularly being rotated to other areas of care [8], such a question is currently only included in the EmONC tool. Other allied and supportive professionals may be a necessary addition to the list of staff cadres, such as biomedical engineers for maintaining equipment and nursing support staff. For all health facility assessment tools, the capacity and readiness of a facility to provide referral to facilities that can provide more complex care for small and sick is a critical indicator of service readiness. The difficulty and inconsistency in measurement of provider skills and training also illustrates the need for further research into human resource tracking, and work to set benchmarks for staffing ratios for neonatal care [8].

Infection prevention and control is essential for all areas of the facility, with newborns particularly vulnerable, and most of the newborn deaths from infections occurring in small babies. The current tools have several general water and sanitation indicators, which should be harmonised across tools to ensure that the basic soap, running water and safe and effective antiseptics are available in labour and delivery and neonatal care areas. A standard indicator that measures whether the newborn space is separate from the paediatric ward, and for whether there is a system for inborn and out born babies could be a potential proxy indicator for service readiness.

### Measuring more complex inpatient care for small and sick newborns

Small and sick babies, especially those born preterm, are at higher risk of multiple childhood morbidities (including visual, hearing and neuro-developmental), with increasing gradient of adverse developmental outcomes by lower gestational age of survivors [41,42]. These newborns often require more complex interventions, such as respiratory support (oxygen, continuous positive airway pressure), treatment of specific complications (feeding, seizures, jaundice), and screening and follow up services (**Figure 1**) [8,43-47]. Many of these interventions carry a risk of harm when not performed with safe equipment or by trained staff. This is illustrated in middle-income settings, where we have seen an increase in impairments among survivors of neonatal care, especially where complex care has been scaled up without due attention to service readiness needs and quality of care [41,42,48].

The existing tools do not capture the large number of items required to deliver complex interventions safely, which would require a facility based monitoring register that also includes process and outcome data (morbidity and mortality) [49]. Such registers have been developed in higher- and middle-income settings [29,49], but are not standardised routine systems. Further research into adaptations of existing tools is an important next step.

Clinical care charts and protocols are essential for quality and safety of neonatal care that requires complex calculations of drug concentrations and specific diluents, dosages, and delivery modes for newborns. In addition to service readiness, the risks of certain interventions can be mitigated by ensuring clinical record keeping, which is known to be sub-standard in many settings [50]. Standardised observation charts for monitoring of vital signs (eg, hourly or three-hourly), fluid input and output, feeding method and volume, and monitoring medications and laboratory tests (eg, serum bilirubin and exchange transfusion thresholds) could support facilities, alongside up-to-date standardised evidence-based guidelines, a list of which is included in the documentation section of our matrix.

### Implications and next steps for monitoring service readiness for inpatient care of small and sick newborns

Amongst existing partners and initiatives, there is widespread recognition of the need to harmonise monitoring systems for perinatal care. The existing EmOC signal functions do not represent the full set of facility-based interventions for mothers and newborns, and small and sick newborns are especially neglected. Given the large number of service readiness requirements for small and sick newborn care, a short list of signal functions for monitoring purposes is a potential solution. Previous work by Gabrysch and colleagues has recommended improvements to these tools [51]. Currently, a global survey led by *Every Newborn* partners, alongside a technical group led by AMDD and UNFPA are working on linking this to the emergency obstetric care indicators, with plans to finalise recommendations for newborn signal functions in 2018-2019 [9].

Periodic evaluation, using health facility surveys, is currently necessary, but ultimately the goal should be to incorporate such assessments into functional and sustainable routine national systems. These should operate independent of donor funding and project mandates. The current health facility assessment tools are costly and time-consuming. Lighter assessments that can be carried out more frequently are also required, and need more research [52]. Even in high-income countries, not all national facilities feed information into one database for national monitoring of inpatient care of small and sick newborns. Low- and middle-income countries that have not moved to electronic information systems have an advantage in that they can leap-frog the situation of having fragmented and discrepant electronic data collection forms which differ from facility to facility or region to region. Exploration of the potential use of DHIS-2 platforms for facility-based monitoring is being carried out as part of the *Every Newborn* metrics work on small and sick newborns [9]. This work supports the growing interest in use of routine health management information systems to monitor aspects of service delivery in facilities [14], and of logistic management information systems (LMIS) to track logistics and supplies.

## CONCLUSIONS

Tracking of service readiness to provide inpatient care of small and sick newborns is needed to gain the required policy attention, accountability and investment that is critical to end preventable newborn deaths, and improve child development. This is reflected in the Global Strategy for Women, Children and Adolescents, the WHO Quality of Care Framework, and is supported by the *Every Newborn* metrics working group. The existing health facility assessments do not generate comparable data, and have very limited assessment of more complex care for small and sick newborns. Indicators in existing tools can be harmonised, but the size and cost of these assessments limits their frequency. Developing a core list of harmonised indicators for use in routine health information systems could help address this gap. Improvements in these monitoring systems are urgently needed to inform efforts to improve quality of care and investments in health systems scale-up, to end preventable newborn death and disability, alongside work to end preventable maternal deaths and stillbirths.
